# Prioritizing debt conversion opportunities for marine conservation

**DOI:** 10.1111/cobi.13540

**Published:** 2020-06-12

**Authors:** Jennifer McGowan, Rob Weary, Leah Carriere, Edward T. Game, Joanna L. Smith, Melissa Garvey, Hugh P. Possingham

**Affiliations:** ^1^ The Nature Conservancy 4245 Fairfax Dr #100 Arlington VA 22203 U.S.A.; ^2^ NatureVest The Nature Conservancy 4245 Fairfax Dr #100 Arlington VA 22203 U.S.A.; ^3^ The Nature Conservancy 48 Montague Road South Brisbane Qld 4101 Australia; ^4^ Nature United The Nature Conservancy 366 Adelaide Street East, Suite 331 Toronto ON M5A 3X9 Canada

**Keywords:** conservation finance, cost‐effectiveness, debt for nature, decision science, prioritization, return on investment, threats, amenazas, ciencias de la decisión, deuda para la naturaleza, financiamiento de la conservación, priorización, rendimiento de la inversión, rentabilidad

## Abstract

Incentivized debt conversion is a financing mechanism that can assist countries with a heavy debt burden to bolster their long‐term domestic investment in nature conservation. The Nature Conservancy, an international conservation‐based nongovernmental organization, is adapting debt conversions to support marine conservation efforts by small island developing states and coastal countries. Prioritizing debt conversion opportunities according to their potential return on investment can increase the impact and effectiveness of this finance mechanism. We developed guidance on how to do so with a decision‐support approach that relies on a novel threat‐based adaptation of cost‐effectiveness analysis. We constructed scenarios by varying parameters of the approach, including enabling conditions, expected benefits, and threat classifications. Incorporating both abatable and unabatable threats affected priorities across planning scenarios. Similarly, differences in scenario construction resulted in unique solution sets for top priorities. We show how environmental organizations, private entities, and investment banks can adopt structured prioritization frameworks for making decisions about conservation finance investments, such as debt conversions. Our guidance can accommodate a suite of social, ecological, and economic considerations, making the approach broadly applicable to other conservation finance mechanisms or investment strategies that seek to establish a transparent process for return‐on‐investment decision‐making.

## Abstract 2

**Article Impact Statement**: Prioritizations that explicitly incorporate abatable and unabatable threats can improve conservation return on investment decision‐making.

## Abstract (es)

Priorización de las Oportunidades de Conversión de Deudas para la Conservación Marina

**Resumen**

La incentivación de la conversión de deudas es un mecanismo financiero que puede apoyar a los países con una gran carga deudora a impulsar su inversión doméstica a largo plazo en la conservación de la naturaleza. The Nature Conservancy, una organización internacional no gubernamental basada en la conservación está adaptando las conversiones de deudas para que apoyen a los esfuerzos de conservación marina realizados por países en desarrollo ubicados en pequeñas islas y por los países costeros. La priorización de las oportunidades de conversión de deudas según su rendimiento potencial de la inversión puede incrementar el impacto y la efectividad de este mecanismo financiero. Desarrollamos una guía de cómo lograr esto con una estrategia de respaldo a las decisiones que depende de una adaptación novedosa basada en amenazas de un análisis de rentabilidad. Construimos escenarios por medio de la variación de los parámetros de alcance (incluyendo a las condiciones de habilitación), los beneficios esperados y las clasificaciones de las amenazas. La incorporación de las amenazas abatibles e imbatibles afectó a las prioridades en todos los escenarios de planeación. De manera similar, las diferencias en la construcción de los escenarios resultaron en un conjunto de soluciones únicas para las principales prioridades. Así demostramos cómo las organizaciones ambientales, entidades privadas y los bancos de inversión pueden adoptar marcos de trabajo de priorización estructurada para la toma de decisiones sobre la inversión en el financiamiento de la conservación, como lo son las conversiones de deudas. Nuestra guía puede satisfacer un conjunto de consideraciones sociales, ecológicas y económicas, lo que vuelve a la estrategia en general pertinente para otros mecanismos de financiamiento de la conservación o estrategias de inversión que busquen establecer un proceso transparente para la toma de decisiones de rendimiento de la inversión.

## Introduction

Incentivized debt conversion is a financing mechanism aimed at assisting countries with heavy debt burdens to bolster domestic investment in development projects, public health, other social programs, and nature conservation. These voluntary transactions typically involve canceling or restructuring a portion of a country's sovereign debt, often with better rates or more favorable repayment terms, in exchange for the country's binding commitment to uphold the conditions of the debt conversion agreement. These agreements typically include measures to deliver positive environmental or social outcomes (Ruiz 2007; UNDP 2017). The first debt conversion for conservation (also known as debt‐for‐nature swaps) occurred in 1987 when Bolivia and an environmental nongovernmental organization (NGO)—Conservation International‐ negotiated the exchange of US$ 650,000 of Bolivian commercial debt for a commitment to conserve 1.5 million ha of forest (Cassimon et al. 2011).

Debt conversions are desirable exchanges for political, economic, and environmental rationales. They make the concept of debt relief politically attractive for creditors, which can be a single country, a consortium of countries (e.g., the Paris Club), or commercial entities, such as banks, to whom countries are indebted. They offer recipient countries an opportunity to reduce their external debt and free up capital to invest in conservation activities (Buckley 2009). The process facilitates greater transparency and structure into conservation‐spending decisions in recipient countries while encouraging broader engagement from civil society in the protection of biodiversity and sustainable use of natural resources—a major driver of NGO support for this mechanism (Ruiz 2007; UNDP 2017).

Throughout the 1990s, other NGOs, including the Rainforest Alliance, The Nature Conservancy (TNC), and the World Wildlife Fund began to play a central role as a third party in negotiating, administering, and implementing debt restructuring for tropical forest conservation (see Shiekh [2018] for a review of past third‐party and bilateral transactions). As of 2018, estimates suggest these third‐party transactions generated the equivalent of US$167 million in local currencies to fund conservation in 16 countries, including Brazil, Costa Rica, Ghana, Madagascar, Mexico, Philippines, and Zambia (Sheikh 2010, 2018).

Although debt conversions peaked in the mid‐1990s (Shiekh 2018), they remain an appealing option for conservation financing. The global economy is experiencing another wave of rapid debt accumulation; debt loads in emerging market and developing economies reached a record high of US$55 trillion in 2018 (Kose et. al. 2020). Many of these countries have limited low‐cost financing options for loan repayment due to poor credit rankings (e.g., Moody's investment grades). The rise of impact investing over the last decade to support meaningful social and environmental causes creates an opportunity to engage private investors in raising the initial capital needed for debt conversion transactions (Pineiro et al. 2018). Changes over the last few decades in the financing instruments available to developing countries and economies in transition means there is more high‐risk, commercial sovereign external debt available to purchase on secondary markets than ever before (UNCTAD 2015). Finally, there is renewed political will for countries to sustainably develop their blue economies through better management and protection of their natural resources driven by commitments to the Convention on Biological Diversity (https://www.cbd.int/sp/targets/) and the UN Sustainable Development Goals (https://sustainabledevelopment.un.org).

Building on these opportunities, TNC extended their involvement in debt conversions by engaging in the first‐ever debt conversion for ocean conservation in 2016. This was a US$21.6 million agreement with the Republic of Seychelles to advance the country's commitment to expand marine protection to 30% of its Exclusive Economic Zone (EEZ); half of this area was allocated to high‐protection conservation zones (Convergence 2017). The Nature Conservancy currently aims to integrate debt conversions as a core component of its marine conservation work to help countries meet their national and international commitments to marine biodiversity protection.

Our intention was to share guidance on how future opportunities for debt conversions may be prioritized with a tailored decision‐support approach (Wall et al. 2017). We devised a prioritization framework that relies on a novel, threat‐based adaptation of cost‐effectiveness analysis that incorporates multiple social, ecological, political, and economic considerations relevant to identifying suitable opportunities for debt conversion. Finally, we applied the approach to 53 small island developing states and coastal nations. We considered these issues through the lens of TNC's experiences and preferences but regard the approach as broadly applicable to other financial mechanisms where there is aim to establish a transparent and structured process for prioritizing conservation investments.

## Overview of the debt Conversion Process

Debt conversions can be conceptualized in 2 stages: pretransaction stage, when the conservation deal is designed and structured, and posttransaction stage, when the agreement is implemented. Here, we focused on the pretransaction stage and acknowledge that what we describe is one representation of a highly contextual and nonlinear process (Deacon & Murphy 1997).

Once a government agrees to consider a debt conversion, TNC works with that government to identify eligible commercial or bilateral debt to target. Depending on which type of debt is targeted, the discount rate of the debt is determined by its market value or by negotiations with the creditor. The Nature Conservancy then raises capital through philanthropic grants, repayable loans from private impact investors, or a combination of both (referred to as blended finance) to support the debt conversion transaction (details on Seychelles transaction in Supporting Information).

Negotiated terms of the transaction will vary by country, and TNC's objectives are to strengthen and expand marine protected area (MPA) policy commitments and support marine spatial planning processes that deliver multiple objectives, including systematically planned, stakeholder‐driven marine protection (Agostini et al. 2015; Jumin et al. 2018). The transaction terms may also fill critical gaps in ecosystem and species representation (Klein et al. 2015), particularly in nearshore habitats that must balance zoning for fisheries management and protected areas (McGowan et al. 2018). Support for the enforcement and monitoring of the existing or updated protected area network is also a priority (Gill et al. 2017).

Given the risk of loan default from the debtor countries, credit enhancement (a process similar to finding a cosignor on a loan for someone with poor credit) must also be secured through a development finance institute such as the World Bank, the U.S. Development Finance Corporation, or private insurers. Aside from addressing transactional risk, the credit enhancement process provides an additional level of oversight because these institutions have stringent guidelines that screen for adverse social, political, and environmental impacts in their project portfolios and provide clear guidance on the types of activities that are prohibited (e.g., population resettlement, unsustainable fishing practices, and child labor [OPIC 2017]).

An essential component of TNC's debt conversion process is to help establish a local, nonprofit, and financially independent conservation trust. The trust is selected through a stakeholder‐driven process and is primarily responsible for administering the newly leveraged conservation funds domestically in the post‐transaction stage. Conservation trusts must have a nongovernment majority on the board to ensure conservation spending aligns with the contractual agreement. The Nature Conservancy maintains a controlling seat on the trust's board for the duration of the term agreement, which is typically 20 years, and activities funded through the trust may include marine habitat restoration; economic diversification, including sustainable fisheries and tourism; ecosystem‐based adaptation to improve community resilience to climate change; and national risk‐reduction plans, among others.

Given the high transaction costs of negotiating a debt conversion process (e.g., financial, legal, political, and conservation planning expertise required), prioritizing opportunities according to their potential return on investment can improve the effectiveness of NGOs, investment banks, and private entities that wish to assist governments through this financial mechanism. We framed our decision‐support strategy around cost‐effectiveness analysis, meaning the expected conservation benefit delivered through an action, multiplied by the probability the action is successful, and divided by the cost of taking the action (Tear et al. 2014).

In conservation practice, cost‐effectiveness analyses are commonly applied to prioritize conservation actions for threatened species recovery (Joseph et al. 2009; Di Fonzo et al. 2017; Martin et al. 2018) and invasive species management (Kerr et al. 2016; Firn et al. 2015) (Supporting Information). Yet cost‐effectiveness analyses are well suited to many other contexts and scales where social, ecological, and economic factors influence how and where constrained resources should be allocated (Tear et al. 2014). This analytic approach is desirable in the case of debt conversions because it allows for nonmonetary values to be quantified as benefits and variations in parameters help develop a range of informative scenarios (Auerbach et al. 2014; Tear et al. 2014). For our purposes, we embed cost‐effectiveness into a 3‐step prioritization framework: first, a screening step in which enabling conditions to filter eligible candidates are identified and compiled; second, a scenario construction step in which the variables of the cost‐effectiveness analysis are defined; and, third, a prioritization step in which a ranked order of priorities for a given scenario is produced and analyzed.

## Application to Small Island Developing States

People living in small island developing states and coastal countries are some of the most vulnerable to the impacts of climate change and natural disasters. They also steward some of the most important fisheries for locally consumed food provisioning (Selig et al. 2018) and important coastal habitats, such as coral reefs (Spalding et al. 2014). When these countries are exposed to tsunamis, earthquakes, hurricanes, and other extreme weather events, their governments are often forced to quickly borrow money for recovery efforts, resulting in unsustainable debt ratios (Harmeling & Eckstein 2013; IMF 2013). These environmental and economic circumstances compromise a government's ability to invest in protecting, managing, and restoring biodiversity, perpetuating their population's vulnerability to natural disasters and impacts from climate change. Thus, small island developing states are a focus of TNC's support for debt conversions and make up the majority of the 53 candidates we considered in this analysis (Supporting Information).

### Incorporating Threats Into Cost‐Effectiveness Prioritizations

A key advantage of using a return‐on‐investment framework is that it allows users to clearly articulate their values, objectives, and constraints through the definition of benefits, costs, and feasibility parameters. We were particularly interested in how threats can be expressed in cost‐effectiveness prioritizations because marine systems are vulnerable to indirect threats, such as heat stress from climate change, and direct threats, such as overfishing and pollution, some of which cannot be abated by common marine management interventions (e.g., establishing protected areas or regulating fishing [Game et al. 2008a, b]). We found that existing examples of cost‐effectiveness analyses do not explicitly accommodate the important distinction between threats that can and cannot be abated by conservation actions (Tulloch et al. 2015). For example, many studies identify abatable threats in order to target relevant management actions so that managers can prioritize across individual or suites of actions that will deliver the largest benefit (e.g., Chades et al. 2015; Auerbach et al. 2014; Firn et al. 2015). In some cases, threats that cannot be directly abated by local management actions are openly excluded from consideration, but acknowledging this decision is rare (Fuentes et al. 2015; Klein et al. 2016).

More direct integration of threats occurs in some analyses through the estimation of the counterfactual (Beher et al. 2016; Firn et al. 2015) –the difference in outcome to what would have happened if no action had been taken (Ferraro & Pattanayak 2006; Joseph et al. 2009)–but these studies do not quantify how unabatable threats influence the estimated value (but see Terrado et al. [2016] and Supporting Information). Because managers rightly want to incorporate risk of failure into their decision‐making (Tulloch et al. 2014), considering diminished effectiveness of an action given the presence of unabatable threats is an important but currently underemphasized aspect of cost‐effectiveness analysis.

It was the desire to account for both abatable and unabatable threats in our prioritization framework that led us to adapt the cost‐effectiveness formula to explicitly account for these different classes of threats. Accordingly, we considered a biodiversity benefit (*B*) that is affected by threats that are abatable (*I_a_*) and unabatable (*I_u_*), depending on the action being implemented, the probability the action succeeds (*P*), and the total cost (*C*) of taking the action according to

(1) $$\mathrm{cost}-\mathrm{effectiveness}\;=\frac{BI_{a}\left(1-\alpha I_{u\;}\right)P}{C},$$

where α is a multiplier that ensures the effect of the unabatable threats does not return a negative value in the overall net benefit function. Therefore, α must be chosen to ensure (1 − α*Iu*) is never negative.

### Defining and compiling enabling conditions

There are many enabling socioeconomic, ecological, governance, and political circumstances that may make a country more or less desirable for engaging in debt conversion. For example, countries that rank poorly on corruption indices may pose too high of a reputational risk for an environmental NGO or a financial institution providing loan guarantees. Further, the desirability of a candidate country may improve if relationships with their government already exist through an NGO's international programs or projects. Not only can existing relationships lower transaction costs, they also help avoid incurring large start‐up costs associated with an organization anchoring in a new country as a result of the debt conversion. Economic indicators are also important, such as the trajectory of the debt to gross domestic product (GDP) ratio, which can signal if a country's economy is stabilizing, because this can affect the willingness of the creditor to offer debt at a discount if the risk of loan default diminishes. The type of debt is another important consideration because concessional loans, which typically offer more favorable repayment conditions than commercial loans, challenge the ability to negotiate a cost differential from the debt conversion that is large enough to provide the incentive for a government to participate. Distinguishing between how indicators and data are used, for example as enabling conditions, parameters in the analysis, or both, requires thoughtful consideration (examples in Table 1).

**Table 1:** Data that are relevant to prioritizing debt conversions and descriptions of their potential uses in the problem definition.^*^

Classes of data	Data type and unit of measure	Enabling conditions	Benefit	Cost	Feasibility
Economic	debt‐to‐GDP ratio (value 0–1)	signals economic stability (>60% may be more likely to consider debt conversion)			higher debt to GDP ratio could mean debt conversion is more feasible.
	risk of debt distress (low‐high)	signals economic stability			higher risk of distress could mean debt conversion is more feasible.
	trajectory of the debt‐to‐GDP ratio (increasing or decreasing)	if decreasing, could signal stabilizing economy and lower risk of loan default for creditors.			if decreasing, could signal debt conversion more infeasible
	investment grade (code)	if low, signals whether countries have limited financing options			if low, could signal a debt conversion more feasible given high‐cost alternative financing
	official development assistance eligibility (yes or no)	signals whether countries meet eligibility for certain grants			
	commercial and bilateral debt amounts ($)	Greater debt can represent opportunity for larger transactions.			greater debt could mean transaction is more feasible.
	type of debt (e.g. concessional versus market loans)	concessional debt challenges the interest rate differential needed for cost savings.		concessional debt likely increases the transaction costs related to raising initial capital.	concessional debt could make it difficult to create incentive large enough for the government to engage.
Governance	sovereignty (yes or no)	may exclude territories from consideration			
	governance indicators (e.g. corruption) (0–1)	if country ranks poorly, could be reputational risk		if country ranks poorly, consider a higher transaction costs.	if country ranks poorly, consider transaction less likely to succeed.
Political	direct access to finance minister (yes or no)	access enables conversation to begin more easily than if no access			
	conservation trust fund (yes or no)	presence of an existing body to serve as conservation trust		if no, transaction cost will be higher to establish trust through stakeholder engagement process	
	organizational presence	if third‐party organization is already present in country, key relationships with stakeholders and decision makers is already established. If third‐party is already present, there are no start‐up costs and lower transaction costs. If third party is already present, improved chance the transaction succeeds. Socioeconomic losses per unit GDP from natural disasters Greater economic vulnerability means greater chance of supporting debt conversion.			
	proportion GDP from Fisheries (%)	signals value of fisheries to economy		if high, could mean greater lost opportunity costs for fishing industries	if high, could mean debt conversion more infeasible because MPAs may be politically undesirable
	fatalities per 100,000 inhabitants from natural disasters	signals population vulnerability			
Ecological	habitat/s (km^2^)	the more habitat in the EEZ, the bigger the opportunity for impact.	amount available		
	EEZ (km^2^)	size of EEZ (potential for minimum area limit)	amount available		
	ecosystem services	do preferred ecosystem services exist in the country?	value of services provided		

* Abbreviations: GDP, gross domestic product; MPA, marine protected area; EEZ, exclusive economic zone.

In an application of TNC's debt conversion prioritization framework, we used a single enabling condition to identify candidates—the debt to GDP ratio of 60% or higher (scenario ≥60%, *n* = 16). This ratio is highly suggestive of unsustainable economic circumstances (IMF 2013) and is a plausible indicator of the potential to purchase debt at a discount and, possibly, favorable conditions for a debtor government to consider debt conversion. To demonstrate the approach, we tested the sensitivity of this enabling condition on the outcomes of the prioritization by varying the ratio to create 4 scenarios of the candidate list. These base scenarios consisted of all candidates (*n* = 53) and those meeting a debt to GDP threshold of ≥40% (*n* = 28) and ≥80% (*n* = 8), in addition to the ≥60% scenario (Supporting Information).

### Scenario Construction

The primary conservation action facilitated by TNC's debt conversion deals is to assist governments in meeting their national goals and international voluntary commitments by expanding marine protection in their exclusive economic zone (EEZ). A particular emphasis is placed on the world's most biodiverse marine habitats, coral reefs. Thus, we constructed scenarios with 2 conservation benefits: total area of the EEZ and total area of coral reef habitat in the EEZ (Supporting Information).

To assign threat estimates, we first derived the mean values per EEZ from 19 normalized data layers available from the updated Human Impacts to Marine Ecosystems database (Halpern et al. 2015). We classified these layers based on Kuempel et al. (2019), which considers all fishing impacts, benthic structures, and direct human impacts, such as shipping, as abatable in relation to MPAs (Supporting Information). Although there is precedent for shipping lanes to be relocated in response to biodiversity concerns (Dransfield et al. 2014), the likelihood of such an effort across our candidate countries is unknown. Thus, we performed additional sensitivity testing on this classification by also treating shipping as an unabatable threat, resulting in a total of 16 scenarios (Supporting Information). We used Jaccard similarity to evaluate how this change in parameter definition and threat classification affected the 10 countries of highest conservation priority across all scenarios (excluding the >80% because there were too few candidates meeting the enabling criteria). Due to the paucity of realistic estimates across candidates for the probability of success and cost parameters, we assigned values of 1, meaning priorities were driven solely by the expected biodiversity benefit in relation to their country‐level threats. We assumed threats are additive and used the smallest scaling parameter possible (α = 0.18) derived from 1/[max (*I_u_*)].

## Results

### Influence of Abatable and Unabatable Threats on Priorities

**Figure 1:**
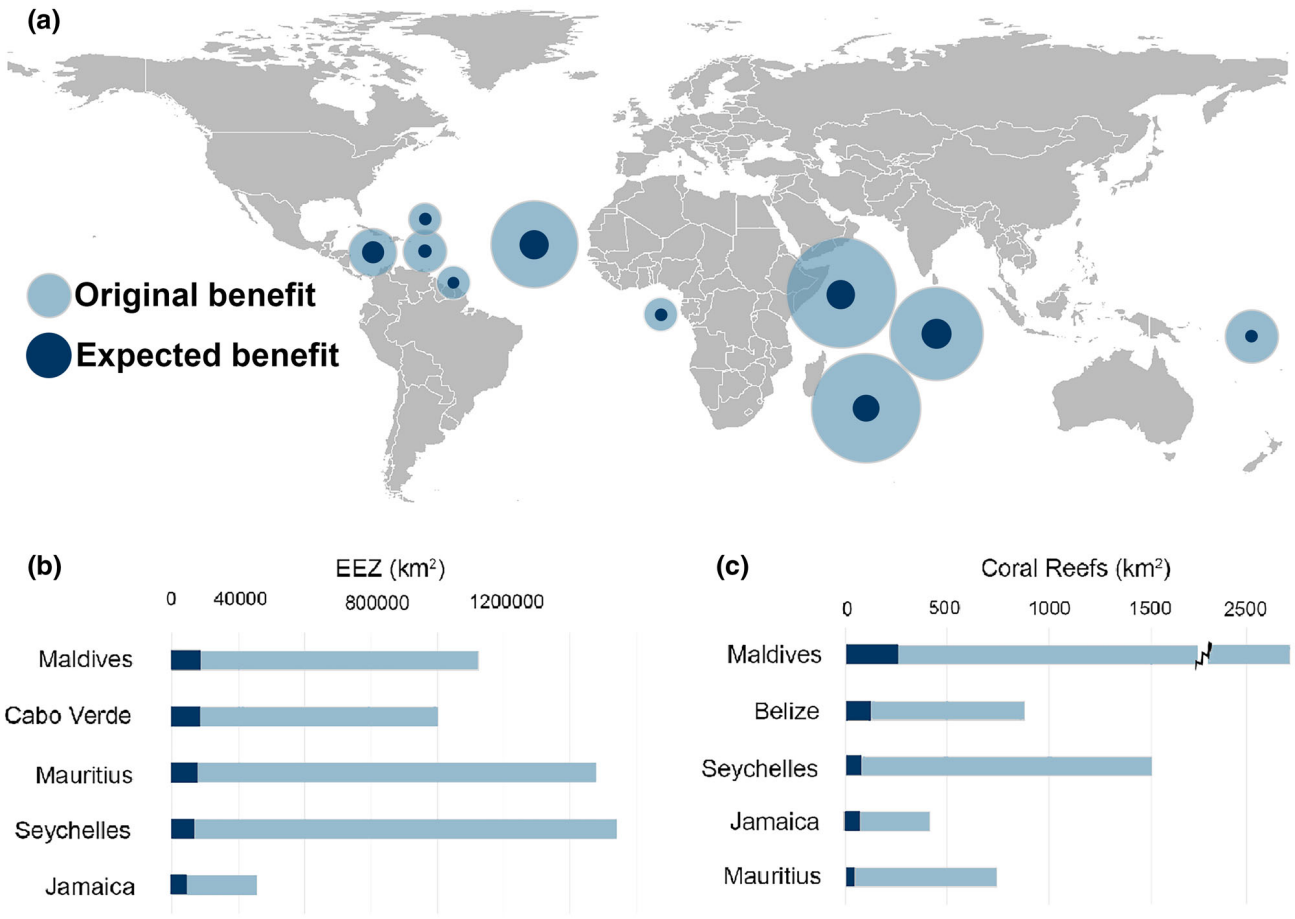
(a) In conservation cost‐effectiveness analysis, effects of inclusion of abatable and unabatable threats to the original benefit for the top‐10 countries based on the size of the Exclusive Economic Zone (EEZ) for the debt to gross domestic product ≥60% scenario. Ranked lists of the top‐5 priority countries under the same scenario based on the expected benefit for (b) area of EEZ and (c) area of coral reefs. See Supporting Information for all prioritization results.

The explicit incorporation of threats into the cost‐effectiveness problem formulation shifts priorities away from those countries that would otherwise simply provide the largest benefit. This is illustrated for the GDP ≥60% scenario, which shows the benefit derived from the EEZ before (original) and after (expected) accounting for threats (Fig 1a). Accounting for threats therefore plays an important role in reshuffling the priority ranking across scenarios. For example, Maldives and Cabo Verde became higher priorities than Mauritius and Seychelles, which had the two largest EEZs of all candidates (Fig 1b). For coral reefs, Belize became a higher priority than Seychelles, despite having a smaller total area of reefs (Fig 1c).

Under our threat classifications, we found more variability between countries with respect to abatable threats than to unabatable threats, which were higher across all regions (Supporting Information). This means the influence of unabatable threats on the expected benefit affected each country more or less equally, reducing the expected benefit of acting in a region by approximately half (mean = 54%), regardless of the benefit used. The variation in abatable threats, therefore, had more influence on the prioritization results for these scenarios at the global scale.

The composition of candidates among the top‐10 priority countries was sensitive to changing enabling conditions and problem definitions (Supporting Information). The final sets showed low (min = 0.05) to moderate (max = 0.69) degrees of overlapping candidates in the top priorities (Supporting Information). Of the 37 countries identified as top priorities across all scenarios, 22% appeared in only one solution set, and 30% appeared in two solution sets. The choices made about enabling conditions and problem definition resulted in very different priorities emerging across scenarios.

## Discussion

Our results showed our prioritization framework can be used to inform investment decisions in debt conversion opportunities to support marine conservation. Identifying and evaluating the financial, political, and economic enabling conditions is the first step in creating a transparent and structured process. Importantly, there are no hard and fast rules for how to use enabling factors in the candidate selection. We encourage the development of a structured decision tree and caution against scoring, weighting, and adding together these factors in arbitrary ways to identify candidates for a prioritization analysis (Game et al. 2013; Brown et al. 2015).

The definition of conservation benefits for these transactions depends on the clear articulation of what is valued by the decision‐makers. Debt conversions for conservation inherently seek to conserve biodiversity but benefits can and should reflect relevant quantifiable ecological, cultural, or social assets deemed important to communities and governments. We used globally available data for the area of the EEZ and mapped coral reefs, but we plan to include other important benefits, such as ecosystem services, as these data become available at the relevant scales.

Our cost‐effectiveness application includes several limitations and caveats. We did not attempt to estimate the operational, management, and transactional costs associated with executing debt conversions across 53 countries. There is only one example of this transaction model from the Republic of Seychelles, making the extrapolation of costs to other contexts purely speculative. For similar reasons, we did not attempt to define a probability of success parameter for debt conversions at a global scale. The primary reasons a debt conversion agreement may fail are through future loan default or misuse of the funds generated from the deal. With only 1 deal completed, for which the post‐transaction stage is still underway (Smith et al. 2019), there are no data on this parameter. However, ensuring credit enhancement and the independence of the conservation trust safeguard against these 2 risks. Another essential risk‐mitigating consideration is to account for governance indicators (Eklund et al. 2011). We encourage political risk or governance indices (e.g., Kaufmann et al. 2010) to be included either as an enabling condition for which a threshold is set to exclude candidates a priori or as a probability of success parameter in future analyses (Table 1). This will be particularly important for prioritizing between countries where region‐specific governance indices exist (Eklund et al. 2011; Tear et al. 2014).

With respect to the explicit consideration of threats in our problem formulation, we considered multiple threats to be additive (as per Kuempel et al. 2019). This approach results in unabatable threats exceeding a value of 1 and subsequently requires an alpha scaling parameter (Eq. 1). In reality, threats interact differently across ecological systems and can be antagonistic (canceling out the impacts of other threats) or synergistic (magnifying threats beyond their additive impacts) (Brown et al. 2013). Accommodating these complex and sophisticated relationships among threats and scaling the accumulation of threats to a value between 0 and 1 to avoid the scaling parameter will require future research. A major advantage for the explicit consideration of abatable and unabatable threats in the problem definition is that it provides a quantitative alternative to expert‐based counterfactual estimation for large‐scale analyses.

We see the development of this approach as a first step toward making robust, repeatable, and transparent decisions about prioritizing conservation finance mechanisms. Applying cost‐effectiveness analyses at global scales requires accepting certain simplifying assumptions, limitations, and uncertainties, yet providing accessible frameworks for decision makers is far more desirable than decisions based solely on opportunism, legacy, or expert opinion (Auerbach et al. 2014). Transparency with governments and stakeholders becomes even more important if the action has been criticized in the past (Cassimon et al. 2011; Silver & Campbell 2018).

Debt conversions are certainly not risk free, and known criticisms must be examined and addressed to ensure success for all parties. First, if the amount of debt converted is marginal relative to the total debt of a country, it may be inadequate to sufficiently improve the state of conservation and deliver anticipated outcomes (Cassimon et al. 2011; Sheikh 2018). Second, given that the terms of the debt transaction are negotiated to meet the conservation objectives of all parties involved, there is a perception that these agreements compromise sovereignty in decision‐making, including over how the conservation funding is allocated to their domestic projects and priorities (Ruiz 2007, UNDP 2017, Silver & Campbell 2018). Third, additional funding for conservation may not be realized if debt conversions are pursued as substitutes for other forms of aid by creditor countries or result in recipient governments adjusting their planned expenditures on conservation and allocate the savings from the debt conversion toward other activities (Cassimon et al. 2011). Finally, quantified evidence of the conservation impact delivered through debt conversions is scarce (Sheik 2018). This reflects a history of poor monitoring and impact evaluation that links the mobilized revenues from the transactions to on‐the‐ground outcomes. Despite these criticisms, debt conversions remain a viable conservation finance mechanism and decades of learning from previous transactions enable safeguards to be put in place to mitigate their documented risks (Cassimon et al. 2011; UNDP 2017).

Addressing questions of how to prioritize finance and conservation investments within a well‐defined framework helps structure resource‐allocation decisions in a rational way. Operationalizing the results of our analysis will be constrained by numerous and varied social, ecological, and political factors that need to be considered for each priority candidate country. Further, the subsequent marine spatial planning processes and spending decisions made by the conservation trusts will require transparent procedures and monitoring to ensure conservation benefits are delivered to nature and people (Giakoumi et al. 2018). The intentional simplicity of this prioritization approach means it can readily accommodate changing enabling factors and serve as an iterative and adaptable decision‐support strategy. We believe the cost‐effectiveness approach described here has wide applicability beyond debt conversions for ocean conservation and can inform other types of conservation challenges where evaluating projects based on their potential return‐on‐investment is desirable for effective decision‐making.

## Supporting information

An example of the debt conversion transaction structure (Appendix S1), review of cost‐effectiveness analysis for conservation (Appendix S2), candidates for debt conversion and their eligibility in relation to the debt to GDP ratio (Appendix S3), data sources for enabling conditions and scenario construction (Appendix S4), classification of threats as abatable or unabatable for scenario treatments (Appendix S5), matrix of scenarios (Appendix S6), example of summed threat class values (Appendix S7), analysis results (Appendix S8.1–S8.4), and Jaccard similarity analysis results (Appendix S9) are available online. The authors are solely responsible for the content and functionality of these materials. Queries (other than absence of the material) should be directed to the corresponding author.
