# Supplementary material for: Prioritizing debt conversion opportunities for marine conservation
Source: Conserv Biol. 2020 Jun 12;34(5):1065–75. doi: 10.1111/cobi.13540 (PMC8129986; doi:10.1111/cobi.13540)
Supplement: Supplementary file 1 — An example of the debt conversion transaction structure (Appendix S1), review of cost‐effectiveness analysis for conservation (Appendix S2), candidates for debt conversion and their eligibility in relation to the debt to GDP ratio (Appendix S3), data sources for enabling conditions and scenario construction (Appendix S4), classification of threats as abatable or unabatable for scenario treatments (Appendix S5), matrix of scenarios (Appendix S6), example of summed threat class values (Appendix S7), analysis results (Appendix S8.1–S8.4), and Jaccard similarity analysis results (Appendix S9) are available online. The authors are solely responsible for the content and functionality of these materials. Queries (other than absence of the material) should be directed to the corresponding author. [file COBI-34-1065-s001.docx]

**Prioritizing debt conversion opportunities for marine conservation**

**­­Supplementary Document**

Appendix S1. Schematic of the transaction of the Seychelles deal and key steps in the structuring process. For more details on the Seychelles debt conversion see Convergence (2017).

Convergence. 2017. Seychelles Debt Conversion for Marine Conservation and Climate Adaptation Case Study. Convergence, Canada. Available from <https://www.convergence.finance/knowledge/3p1S3pSTVKQYYC2ecwaeiK/view> (accessed August 2019).

Appendix S2. A mini-review of cost-effectiveness analysis for conservation applications

We reviewed a sample of the published literature to examine how cost-effectiveness analysis (CEA) has been applied to support conservation planning and prioritization. Web of Science was searched in June 2019 with the following search terms: TS=(conservation AND planning AND spatial AND (("cost-effect*" OR "cost effect*") OR priorit* OR marxan OR zonation)). This was refined by **PUBLICATION YEARS:** (2019 OR 2018 OR 2017 OR 2016 OR 2015) AND **WEB OF SCIENCE CATEGORIES:** ( BIODIVERSITY CONSERVATION ) AND **DOCUMENT TYPES:** (ARTICLE ).

The search resulted in 44 papers plus an additional reference was added to this review from the literature cited in the main manuscript (Tear et al. 2014) for a total of 45 papers. The papers were then reviewed and separated into three categories. The first category was for papers that used a cost-effectiveness approach as defined by the following: CEA = Benefit/Cost; or CEA = Benefit*Feasibility/Cost. The second category was for papers that used an optimization or algorithmic approach to identify cost-efficient solutions to a well-defined problem, for example using Marxan, Zonation or a custom algorithm to optimize solutions. The third category was for papers that were not relevant because they either did not apply a prioritization approach, were a synthesis or review paper, or a perspective piece.

| **Cost-effectiveness analysis** | **Optimizations or algorithmic approaches** | **No prioritization or not relevant**  **(e.g. review paper or opinion piece)** |
| --- | --- | --- |
| (*1*–*18*) | (*19*–*34*) | (*35*–*45*) |

Of the analyses that used cost-effectiveness to prioritize conservation actions, 61% were applied to terrestrial conservation challenges, with the remaining applications directed at terrestrial and freshwater systems (17%), terrestrial and marine systems (17%), and one study spanning all three systems (5%).

The benefits defined considered single threatened species (17%), multiple threatened species (33%), ecosystem services (17%), biodiversity indices (5%) with the remaining studies applied to management outcomes for invasive species (28%).

The vast majority of these studies used cost-effectiveness to prioritize across multiple actions (83%) with the remaining prioritizing the placement of a single action across a study region. Nearly half of the studies reviewed estimated a counterfactual (44%), and the majority of those studies (5 out of 8), looked at species focused threat management prioritizations. The only study that explicitly accounted for both abatable and unabatable threats in the estimation of the counterfactual was Terrado et al. (2016).

Of those studies using optimization approaches 75% used Marxan (N=9) or Zonation (N=1), and the remaining used custom algorithms to optimize action/s for conservation in cost-efficient ways. We did not systematically review these studies as we were primarily concerned with how cost-effectiveness analysis is used to prioritize conservation actions and not reserve design analyses.

List of References:

1. Auerbach N, Tulloch AI, Possingham HP. 2014. Informed actions: where to cost effectively manage multiple threats to species to maximize return on investment. Ecological Applications **24:** 1357-1373.
2. Tear TH, Stratton BN, Game ET, Brown MA, Apse CD, Shirer RR. 2014. A return-on-investment framework to identify conservation priorities in Africa. Biological Conservation. **173**: 42-52. DOI: 10.1016/j.biocon.2014.01.028.
3. Martin et al. 2019. Prioritizing recovery funding to maximize conservation of endangered species. Nature Ecology and Evolution. **3** (1) DOI: 10.1038/s41559-018-0743-8.
4. Roberts M, Cresswell W, Hanley N. 2018. Prioritising Invasive Species Control Actions: Evaluating Effectiveness, Costs, Willingness to Pay and Social Acceptance. Ecological Economics. **152**. DOI: 10.1016/j.ecolecon.2018.05.027.
5. Klein CJ, Beher J, Chaloupka M, Hamann M, Limpus C, Possingham HP. Prioritization of Marine Turtle Management Projects: A Protocol that Accounts for Threats to Different Life History Stages. Conservation Letters. **10** (5) DOI: 10.1111/conl.12324
6. Saunders et al. 2017. Simple rules can guide whether land- or ocean-based conservation will best benefit marine ecosystems. PLOS Biology. **15**(9) DOI: 10.1371/journal.pbio.2001886.
7. Wang YY, Atallah S, Shao GF. 2017. Spatially explicit return on investment to private forest conservation for water purification in Indiana, USA. Ecosystem Services. **26** DOI: 10.1016/j.ecoser.2017.06.004.
8. Drechsler M, Smith HG, Sturm A, Watzold F. 2016. Cost-effectiveness of conservation payment schemes for species with different range sizes. Conservation Biology. **30** (4) DOI: 10.1111/cobi.12708.
9. Terrado M, Momblanch A, Bardina M, Boithias L, Munne A, Sabater S, Solera A, Acuna V. 2016. Integrating ecosystem services in river basin management plans. Journal of Applied Ecology. **53** (3) DOI: 10.1111/1365-2664.12613.
10. Beher J, Possingham HP, Hoobin S, Dougall C, Klein C. 2016. Prioritising catchment management projects to improve marine water quality. Environmental Science & Policy. DOI: 10.1016/j.envsci.2016.02.005.
11. Rose LE, Heard GW, Chee YE, Wintle BA. 2016. Cost-effective conservation of an endangered frog under uncertainty. Conservation Biology. **30** (2) DOI: 10.1111/cobi.12626.
12. Kerr NZ, Baxter PWJ, Salguero-Gomez R, Wardle GM, Buckley YM. 2016. Prioritizing management actions for invasive populations using cost, efficacy, demography and expert opinion for 14 plant species world-wide. Journal of Applied Ecology. **53** (2) DOI: 10.1111/1365-2664.12592.
13. McConnachie MM, van Wilgen BW, Ferraro PJ, Forsyth AT, Richardson DM, Gaertner M, Cowling RM. 2016. Using counterfactuals to evaluate the cost-effectiveness of controlling biological invasions. Ecological Applications.  **26** (2) DOI: 10.1890/15-0351.
14. Firn J et al. 2015. Priority threat management of invasive animals to protect biodiversity under climate change. Global Change Biology. **21** (11) DOI: 10.1111/gcb.13034
15. Firn J, Martin TG, Chades I, Walters B, Hayes J, Nicol S, Carwardine J. 2015. Priority threat management of non-native plants to maintain ecosystem integrity across heterogeneous landscapes. Journal of Applied Ecology.  **52** (5) DOI: 10.1111/1365-2664.12500.
16. Austin Z, McVittie A, McCracken D, Moxey A, Moran D, White PCL. 2015. Integrating quantitative and qualitative data in assessing the cost-effectiveness of biodiversity conservation programmes. Biodiversity and Conservation. **24** (6) DOI: 10.1007/s10531-015-0861-4.
17. Tulloch AIT, Maloney RF, Joseph LN, Bennett JR, Di Fonzo MMI, Probert WJM, O'Connor SM, Densem JP, Possingham HP. 2015. Effect of risk aversion on prioritizing conservation projects. Conservation Biology. **29** (2) DOI: 10.1111/cobi.12386.
18. Chades I, Nicol S, van Leeuwen S, Walters B, Firn J, Reeson A, Martin TG, Carwardine J. 2015. Benefits of integrating complementarity into priority threat management. **29** (2) DOI: 10.1111/cobi.12413.
19. Klingbeil BT et al. 2018. Evaluating a focal-species approach for tidal marsh bird conservation in the northeastern United States. Condor. **120** (4) DOI: 10.1650/CONDOR-18-88.1.
20. Boon PY, Beger M. 2016. The effect of contrasting threat mitigation objectives on spatial conservation priorities. Marine Policy. **68** DOI: 10.1016/j.marpol.2016.02.010.
21. Magris RA, Pressey RL, Mills M, Vila-Nova DA, Floeter S. 2017. Integrated conservation planning for coral reefs: Designing conservation zones for multiple conservation objectives in spatial prioritisation. Global Ecology & Conservation. **11** DOI: 10.1016/j.gecco.2017.05.002.
22. Hermoso V, Cattarino L, Kennard MJ, Watts M, Linke S. 2015. Catchment zoning for freshwater conservation: refining plans to enhance action on the ground. Journal of Applied Ecology. **52** (4) DOI: 10.1111/1365-2664.12454.
23. Li Yong D, Barton PS, Ikin K, Evans MJ, Crane M, Okada S, Cunningham SA, Lindenmayer DB. 2018. Cross-taxonomic surrogates for biodiversity conservation in human-modified landscapes - A multi-taxa approach. Biological Conservation. **224** DOI: 10.1016/j.biocon.2018.06.008.
24. Giakoumi S, Brown CJ, Katsanevakis S, Saunders MI, Possingham HP. 2015. Using threat maps for cost-effective prioritization of actions to conserve coastal habitats. Marine Policy. **61** DOI: 10.1016/j.marpol.2015.07.004.
25. Kujala H, Lahoz-Monfort JJ, Elith J, Moilanen A. 2018. Not all data are equal: Influence of data type and amount in spatial conservation prioritisation. Methods in Ecology and Evolution. **9** (11) DOI: 10.1111/2041-210X.13084.
26. Tulloch VJ, Klein CJ, Jupiter SD, Tulloch AIT, Roelfsema C, Possingham HP. 2017. Trade-offs between data resolution, accuracy, and cost when choosing information to plan reserves for coral reef ecosystems. Journal of Environmental Management. **188** DOI: 10.1016/j.jenvman.2016.11.070.
27. Evans MC, Tulloch AIT, Law EA, Raiter KG, Possingham HP, Wilson KA. 2015. Clear consideration of costs, condition and conservation benefits yields better planning outcomes. Biological Conservation. **191** DOI: 10.1016/j.biocon.2015.08.023.
28. Cattarino L, Hermoso V, Bradford LW, Carwardine J, Wilson KA, Kennard MJ, Linke S. 2016. Accounting for continuous species' responses to management effort enhances cost-effectiveness of conservation decisions. Biological Conservation. **197** DOI: 10.1016/j.biocon.2016.02.030.
29. Molin PG, Chazdon R, Ferraz SFD, Brancalion PHS. 2018. A landscape approach for cost-effective large-scale forest restoration. Journal of Applied Ecology. **55** (6) DOI: 10.1111/1365-2664.13263.
30. Langhans SD, Gessner J, Hermoso V, Wolter C. 2016. Coupling systematic planning and expert judgement enhances the efficiency of river restoration. Science of the Total Environment. **560** DOI: 10.1016/j.scitotenv.2016.03.232.
31. Fuentes MMPB et al. 2015. A decision framework for prioritizing multiple management actions for threatened marine megafauna. Ecological Applications. **25**(1) DOI: 10.1890/13-1524.1.
32. Strassburg BBN et al. 2019. Strategic approaches to restoring ecosystems can triple conservation gains and halve costs. Nature Ecology and Evolution. **3** (1) DOI: 10.1038/s41559-018-0743-8.
33. Dhanjal-Adams KL, Mustin K, Possingham HP, Fuller RA. 2016. Optimizing disturbance management for wildlife protection: the enforcement allocation problem. Journal of Applied Ecology.  **53** (4) DOI: 10.1111/1365-2664.12606.
34. Neeson TM, Moody AT, O'Hanley JR, Diebel M, Doran PJ, Ferris MC, Colling T, McIntyre, PB. 2018. Aging infrastructure creates opportunities for cost-efficient restoration of aquatic ecosystem connectivity. Ecological Applications.  **28** (6) DOI: 10.1002/eap.1750.
35. Caro, T. 2016. Guidelines for wildlife monitoring: savannah herbivores. Tropical Conservation Science. **(9)** 1: DOI: 10.1177/194008291600900102.
36. Varela E. 2018. Promoting biodiversity values of small forest patches in agricultural landscapes: Ecological drivers and social demand. Science of the Total Environment. **619** DOI: 10.1016/j.scitotenv.2017.11.190.
37. Weise FJ, Hayward MW, Aguirre RC, Tomeletso M, Gadimang P, Somers MJ, Stein AB. 2018. Size, shape and maintenance matter: A critical appraisal of a global carnivore conflict mitigation strategy - Livestock protection kraals in northern Botswana. Biological Conservation. **225** DOI: 10.1016/j.biocon.2018.06.023.
38. Tan YM, Saunders JE, Yaakub SM. 2018. A proposed decision support tool for prioritising conservation planning of Southeast Asian seagrass meadows: combined approaches based on ecosystem services and vulnerability analyses. Botanica Marina. **61** (3) DOI: 10.1515/bot-2017-0117.
39. Tanentzap AJ, Walker S, Stephens RTT. 2017. Better practices for reporting on conservation. Conservation Letters. **10** (1) DOI: 10.1111/conl.12229.
40. Pe'er G et al. 2017. Adding Some Green to the Greening: Improving the EU's Ecological Focus Areas for Biodiversity and Farmers Conservation Letters. **10**(5) DOI: 10.1111/conl.12333.
41. Tapley, B; Bradfield, KS; Michaels, C; Bungard, M. 2015. Amphibians and conservation breeding programmes: do all threatened amphibians belong on the ark? Biodiversity and Conservation. **24** (11) DOI: 10.1007/s10531-015-0966-9.
42. Roberge JM, Lamas T, Lundmark T, Ranius T, Felton A, Nordin A. 2015. Relative contributions of set-asides and tree retention to the long-term availability of key forest biodiversity structures at the landscape scale. Journal of Environmental Management. **154** DOI: 10.1016/j.jenvman.2015.02.040
43. McNamara L, Jones C. 2016. Improving investment decision-making in community-based conservation. Australasian Journal of Environmental Management. **23** (4) DOI: 10.1080/14486563.2016.1214929.
44. Ruffino L, Zarzoso-Lacoste D, Vidal E. 2015. Assessment of invasive rodent impacts on island avifauna: methods, limitations and the way forward. Wildlife Research. **42** (1) DOI: 10.1071/WR15047.
45. Carwardine J, Martin TG, Firn J, Reyes RP, Nicol S, Reeson A, Grantham HS, Stratford D, Kehoe L, Chades I. 2019. Priority Threat Management for biodiversity conservation: A handbook. Journal of Applied Ecology. **56** (2) DOI: 10.1111/1365-2664.13268.

Appendix S3. Candidates for debt conversion and their eligibility in relation to the Debt to GDP ratio.

| **Country or territory** | **All candidates considered**  (N=53) | **Debt to GDP ratio at 40% or higher**  (N= 28) | **Debt to GDP ratio at 60% or higher**  (N=16) | **Debt to GDP ratio at 80% or higher**  (N=8) |
| --- | --- | --- | --- | --- |
| American Samoa | ✓ |  |  |  |
| Anguilla | ✓ |  |  |  |
| Antigua & Barbuda | ✓ | ✓ | ✓ | ✓ |
| Bahamas | ✓ | ✓ |  |  |
| Barbados | ✓ | ✓ | ✓ | ✓ |
| Belize | ✓ | ✓ | ✓ | ✓ |
| British Virgin Islands | ✓ |  |  |  |
| Cabo Verde | ✓ | ✓ | ✓ | ✓ |
| Cayman Islands | ✓ |  |  |  |
| Comoros | ✓ |  |  |  |
| Cook Islands | ✓ |  |  |  |
| Cuba | ✓ | ✓ |  |  |
| Dominica | ✓ | ✓ | ✓ | ✓ |
| Dominican Republic | ✓ |  |  |  |
| Federated States of Micronesia | ✓ |  |  |  |
| Fiji | ✓ | ✓ |  |  |
| French Polynesia | ✓ |  |  |  |
| Grenada | ✓ | ✓ | ✓ |  |
| Guadeloupe | ✓ |  |  |  |
| Guam | ✓ |  |  |  |
| Guinea-Bissau | ✓ | ✓ |  |  |
| Guyana | ✓ | ✓ |  |  |
| Haiti | ✓ |  |  |  |
| Jamaica | ✓ | ✓ | ✓ | ✓ |
| Kiribati | ✓ |  |  |  |
| Maldives | ✓ | ✓ | ✓ |  |
| Marshall Islands | ✓ |  |  |  |
| Martinique | ✓ |  |  |  |
| Mauritius | ✓ | ✓ | ✓ |  |
| Monserrat | ✓ |  |  |  |
| Netherland Antilles | ✓ |  |  |  |
| New Caledonia | ✓ |  |  |  |
| Niue | ✓ |  |  |  |
| Nauru | ✓ | ✓ | ✓ |  |
| Northern Marianas | ✓ |  |  |  |
| Palau | ✓ |  |  |  |
| Papua New Guinea | ✓ |  |  |  |
| Puerto Rico | ✓ | ✓ |  |  |
| Saint Kitts & Nevis | ✓ | ✓ | ✓ |  |
| Saint Lucia | ✓ | ✓ | ✓ |  |
| Saint Vincent & the Grenadines | ✓ | ✓ | ✓ | ✓ |
| Samoa | ✓ | ✓ |  |  |
| Sao Tome & Principe | ✓ | ✓ | ✓ | ✓ |
| Seychelles | ✓ | ✓ | ✓ |  |
| Solomon Islands | ✓ |  |  |  |
| Suriname | ✓ | ✓ | ✓ |  |
| Timor-Leste | ✓ |  |  |  |
| Tonga | ✓ | ✓ |  |  |
| Trinidad & Tobago | ✓ | ✓ |  |  |
| Turks & Caicos | ✓ |  |  |  |
| Tuvalu | ✓ | ✓ |  |  |
| United States Virgin Islands | ✓ | ✓ |  |  |
| Vanuatu | ✓ | ✓ |  |  |

Appendix S4. Data sources for enabling conditions and scenario construction.

| **Data Layer** | **Source** | **Treatment per EEZ** | **References** |
| --- | --- | --- | --- |
| Coral Reefs (km2) | WCMC 2010 | Sum | <http://data.unep-wcmc.org/datasets/1> |
| Exclusive Economic Zones 200nm v10 (km2) | Flanders Marine Institute 2018 | Area | http://www.marineregions.org/ |
| Debt to GDP ratios | Central Intelligence Agency | *NA* | https://www.cia.gov/library/publications/the-world-factbook/rankorder/2186rank.html |
| Threats | Human Impacts to Marine Ecosystems | Mean values of each threat | Halpern et al. 2015 [www.nceas.ucsb.edu/globalmarine](http://www.nceas.ucsb.edu/globalmarine) |

Appendix S5. Classification of threats as abatable or unabatable in relation to marine protected areas. Treatment 1 follows the classification of Kuempel et al. (2019) while Treatment 2 acts as a sensitivity test for the classification of shipping.

| **Human Activity** | **Treatment 1: Shipping as abatable** | **Treatment 2: Shipping as unabatable** |
| --- | --- | --- |
| Artisanal fishing | **✓** | **✓** |
| Demersal destructive fishing | **✓** | **✓** |
| Demersal non-destructive fishing high bycatch | **✓** | **✓** |
| Demersal non-destructive fishing low bycatch | **✓** | **✓** |
| Oil rigs | **✓** | **✓** |
| Pelagic high bycatch | **✓** | **✓** |
| Pelagic low bycatch | **✓** | **✓** |
| Shipping | **✓** | ✖ |
| Inorganic nutrients | ✖ | ✖ |
| Invasive species | ✖ | ✖ |
| Night lights | ✖ | ✖ |
| Ocean acidification | ✖ | ✖ |
| Ocean pollution | ✖ | ✖ |
| Plumes fertilizer | ✖ | ✖ |
| Plumes pesticides | ✖ | ✖ |
| Population | ✖ | ✖ |
| Sea level rise | ✖ | ✖ |
| Sea surface temperature | ✖ | ✖ |
| UV | ✖ | ✖ |
| **✓ =** abatable ✖ = unabatable | | |

Appendix S6. Matrix of 16 scenario constructions and names based on enabling conditions, benefits, and threat treatments.

|  | **Enabling Conditions** | | | |
| --- | --- | --- | --- | --- |
| **Benefits** | All | GDP 40 | GDP 60 | GDP 80 |
| **Threat Treatment 1- shipping as abatable** | | | | |
| **EEZ** | All _EEZ_T1 | GDP_40_EEZ_T1 | GDP_60_EEZ_T1 | GDP 80_EEZ_T1 |
| **Coral** | All _Coral_T1 | GDP_40_Reef_T1 | GDP_60_Reefs_T1 | GDP 80_Coral Reefs_T1 |
| **Threat Treatment 20 shipping as unabatable** | | | | |
| **EEZ** | All _EEZ_T2 | GDP_40_EEZ_T2 | GDP_60_EEZ_T2 | GDP 80_EEZ_T2 |
| **Coral** | All _Coral_T2 | GDP_40_Reef_T2 | GDP_60_Reefs_T2 | GDP 80_Coral Reefs_T2 |


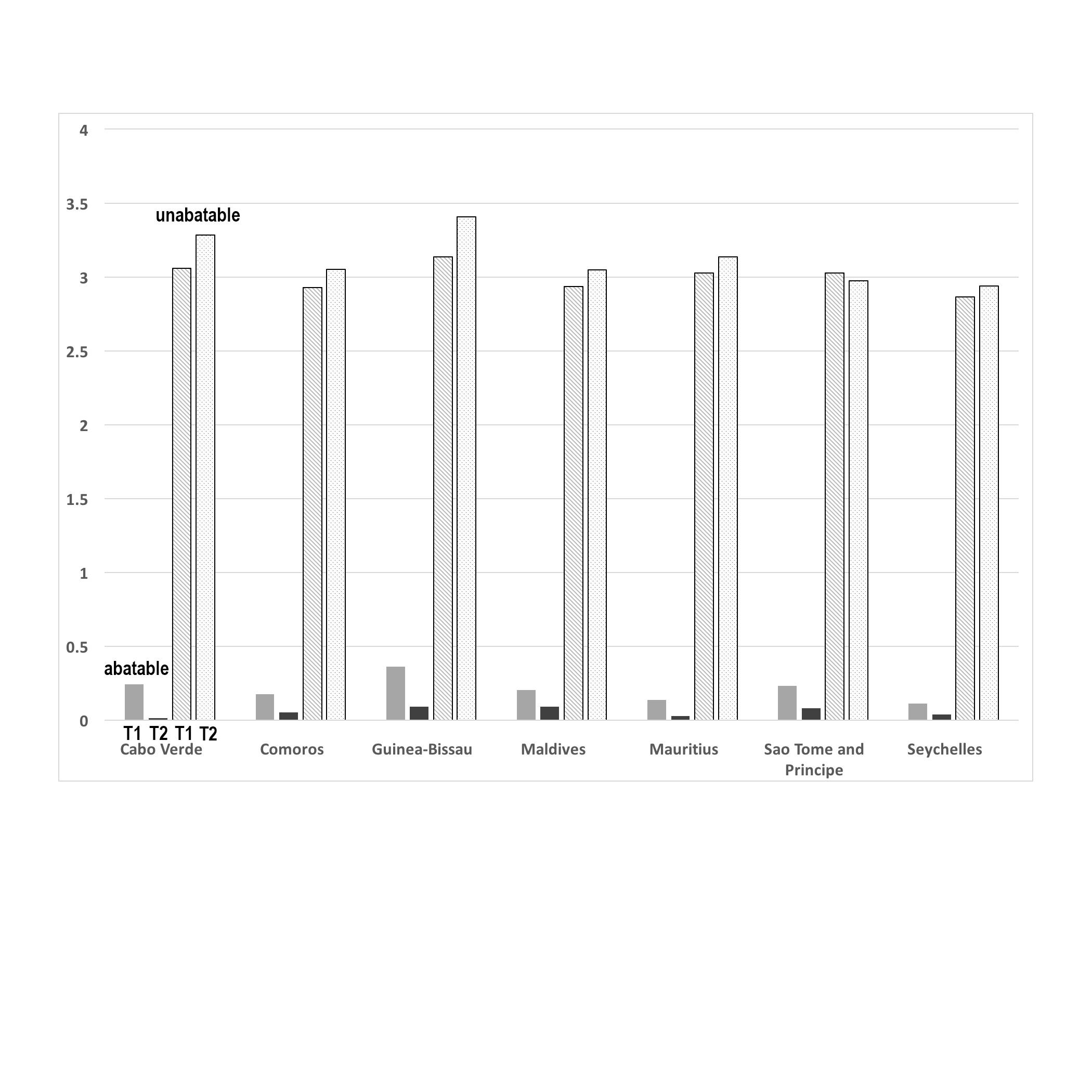


Appendix S7. Example of the total abatable and unabatable threats as they vary across a sample of candidate countries. The sensitivity of the threat values to the classification of shipping as abatable or unabatable can be observed in the difference between threat treatment 1 (T1) and threat treatment 2 (T2).

­

|  | Appendix S8.1 Results from scenarios which consider “All” candidates | | | | |
| --- | --- | --- | --- | --- | --- |
| **Rank** | **All _EEZ_T1** | **All _EEZ_T2** | **All_Reef_T1** | **All_Reef_T2** | |
| 1 | Papua New Guinea | Papua New Guinea | Papua New Guinea | Papua New Guinea | |
| 2 | Federated States of Micronesia | Federated States of Micronesia | Cuba | Maldives | |
| 3 | French Polynesia | Solomon Islands | Bahamas | Solomon Islands | |
| 4 | Bahamas | Northern Marianas | New Caledonia | Cuba | |
| 5 | Northern Marianas | Kiribati | Maldives | Federated States of Micronesia | |
| 6 | Kiribati | Maldives | Federated States of Micronesia | Belize | |
| 7 | Solomon Islands | Marshall Islands | Solomon Islands | Bahamas | |
| 8 | Maldives | Seychelles | Fiji | Fiji | |
| 9 | Cabo Verde | Palau | Belize | Marshall Islands | |
| 10 | Marshall Islands | Mauritius | French Polynesia | Seychelles | |
| 11 | Mauritius | Cuba | Marshall Islands | Kiribati | |
| 12 | New Caledonia | Fiji | Seychelles | Palau | |
| 13 | Seychelles | Nauru | Jamaica | Tuvalu | |
| 14 | Cuba | Tuvalu | Kiribati | Haiti | |
| 15 | Dominican Republic | Guam | Netherland Antilles | Mauritius | |
| 16 | Fiji | Bahamas | Dominican Republic | Netherland Antilles | |
| 17 | Cook Islands | American Samoa | Haiti | Samoa | |
| 18 | Palau | Dominican Republic | Mauritius | Jamaica | |
| 19 | Jamaica | Timor-Leste | Palau | Dominican Republic | |
| 20 | Puerto Rico | Vanuatu | Tonga | Vanuatu | |
| 21 | Vanuatu | French Polynesia | Turks and Caicos | Northern Marianas | |
| 22 | Guam | Sao Tome and Principe | Vanuatu | Guam | |
| 23 | Tonga | Samoa | Puerto Rico | Comoros | |
| 24 | Caymen Islands | Cabo Verde | British Virgin Islands | Tonga | |
| 25 | Guyana | Jamaica | Caymen Islands | New Caledonia | |
| 26 | American Samoa | Guinea-Bissau | Tuvalu | Turks and Caicos | |
| 27 | Barbados | Comoros | Comoros | Guadeloupe | |
| 28 | Tuvalu | Cook Islands | Guam | French Polynesia | |
| 29 | Haiti | Haiti | Northern Marianas | Puerto Rico | |
| 30 | Turks and Caicos | Tonga | Guadeloupe | Timor-Leste | |
| 31 | Netherland Antilles | Puerto Rico | Samoa | Martinique | |
| 32 | Guinea-Bissau | Guadeloupe | Martinique | Antigua and Barbuda | |
| 33 | Antigua and Barbuda | Guyana | Saint Kitts and Nevis | Saint Lucia | |
| 34 | Nauru | Antigua and Barbuda | Antigua and Barbuda | British Virgin Islands | |
| 35 | British Virgin Islands | Suriname | Grenada | American Samoa | |
| 36 | Sao Tome and Principe | Netherland Antilles | Cook Islands | Saint Kitts and Nevis | |
| 37 | Anguilla | Turks and Caicos | United States Virgin Islands | United States Virgin Islands | |
| 38 | Niue | Martinique | Saint Vincent and the Grenadines | Dominica | |
| 39 | Comoros | Belize | Saint Lucia | Trinidad and Tobago | |
| 40 | Suriname | Trinidad and Tobago | Trinidad and Tobago | Cook Islands | |
| 41 | Trinidad and Tobago | New Caledonia | Timor-Leste | Saint Vincent and the Grenadines | |
| 42 | Guadeloupe | Dominica | Anguilla | Grenada | |
| 43 | Timor-Leste | Niue | Barbados | Nauru | |
| 44 | Martinique | Anguilla | Dominica | Monserrat | |
| 45 | United States Virgin Islands | United States Virgin Islands | American Samoa | Anguilla | |
| 46 | Samoa | Monserrat | Niue | Caymen Islands | |
| 47 | Saint Vincent and the Grenadines | Barbados | Monserrat | Barbados | |
| 48 | Dominica | British Virgin Islands | Nauru | Niue | |
| 49 | Belize | Saint Lucia |  |  | |
| 50 | Grenada | Saint Vincent and the Grenadines | |  | |
| 51 | Saint Lucia | Grenada | |  |  |
| 52 | Monserrat | Caymen Islands | |  |  |
| 53 | Saint Kitts and Nevis | Saint Kitts and Nevis | |  |  |

| Appendix S8.2. Results from scenarios which consider “GDP 40” candidates |
| --- |

| **Rank** | **GDP_40_EEZ_T1** | **GDP_40_EEZ_T2** | **GDP_40_Reef_T1** | **GDP_40_Reef_T2** |
| --- | --- | --- | --- | --- |
| 1 | Bahamas | Maldives | Cuba | Maldives |
| 2 | Maldives | Seychelles | Bahamas | Cuba |
| 3 | Cabo Verde | Mauritius | Maldives | Belize |
| 4 | Mauritius | Cuba | Fiji | Bahamas |
| 5 | Seychelles | Fiji | Belize | Fiji |
| 6 | Cuba | Tuvalu | Seychelles | Seychelles |
| 7 | Fiji | Bahamas | Jamaica | Tuvalu |
| 8 | Jamaica | Timor-Leste | Mauritius | Mauritius |
| 9 | Puerto Rico | Vanuatu | Tonga | Samoa |
| 10 | Vanuatu | Sao Tome and Principe | Vanuatu | Jamaica |
| 11 | Tonga | Samoa | Puerto Rico | Vanuatu |
| 12 | Guyana | Cabo Verde | Tuvalu | Tonga |
| 13 | Barbados | Jamaica | Samoa | Puerto Rico |
| 14 | Tuvalu | Guinea-Bissau | Saint Kitts and Nevis | Timor-Leste |
| 15 | Guinea-Bissau | Tonga | Antigua and Barbuda | Antigua and Barbuda |
| 16 | Antigua and Barbuda | Puerto Rico | Grenada | Saint Lucia |
| 17 | Sao Tome and Principe | Guyana | United States Virgin Islands | Saint Kitts and Nevis |
| 18 | Suriname | Antigua and Barbuda | Saint Vincent and the Grenadines | United States Virgin Islands |
| 19 | Trinidad and Tobago | Suriname | Saint Lucia | Dominica |
| 20 | Timor-Leste | Belize | Trinidad and Tobago | Trinidad and Tobago |
| 21 | United States Virgin Islands | Trinidad and Tobago | Timor-Leste | Saint Vincent and the Grenadines |
| 22 | Samoa | Dominica | Barbados | Grenada |
| 23 | Saint Vincent and the Grenadines | United States Virgin Islands | Dominica | Barbados |
| 24 | Dominica | Barbados |  |  |
| 25 | Belize | Saint Lucia |  |  |
| 26 | Grenada | Saint Vincent and the Grenadines |  |  |
| 27 | Saint Lucia | Grenada |  |  |
| 28 | Saint Kitts and Nevis | Saint Kitts and Nevis |  |  |

Appendix S8.3. Results from scenarios which consider “GDP 60” candidates

| **Rank** | | **GDP_60_EEZ_T1** | **GDP_60_EEZ_T2** | **GDP_60_Reefs_T1** | **GDP_60_Reefs_T2** |
| --- | --- | --- | --- | --- | --- |
| 1 | Maldives | | Maldives | Maldives | Maldives |
| 2 | Cabo Verde | | Seychelles | Belize | Belize |
| 3 | Mauritius | | Mauritius | Seychelles | Seychelles |
| 4 | Seychelles | | Nauru | Jamaica | Mauritius |
| 5 | Jamaica | | Sao Tome and Principe | Mauritius | Jamaica |
| 6 | Barbados | | Cabo Verde | Saint Kitts and Nevis | Antigua and Barbuda |
| 7 | Antigua and Barbuda | | Jamaica | Antigua and Barbuda | Saint Lucia |
| 8 | Nauru | | Antigua and Barbuda | Grenada | Saint Kitts and Nevis |
| 9 | Sao Tome and Principe | | Suriname | Saint Vincent and the Grenadines | Dominica |
| 10 | Suriname | | Belize | Saint Lucia | Saint Vincent and the Grenadines |
| 11 | Saint Vincent and the Grenadines | | Dominica | Barbados | Grenada |
| 12 | Dominica | | Barbados | Dominica | Nauru |
| 13 | Belize | | Saint Lucia | Nauru | Barbados |
| 14 | Grenada | | Saint Vincent and the Grenadines |  |  |
| 15 | Saint Lucia | | Grenada |  |  |
| 16 | Saint Kitts and Nevis | | Saint Kitts and Nevis |  |  |

Appendix S8.4. Results from scenarios which consider “GDP 80” candidates

| **Rank** | | **GDP 80_EEZ_T1** | **GDP 80_EEZ_T2** | **GDP 80_Reefs_T1** | **GDP 80_Reefs_T2** |
| --- | --- | --- | --- | --- | --- |
| 1 | Cabo Verde | | Fiji | Fiji | Belize |
| 2 | Jamaica | | Sao Tome and Principe | Belize | Fiji |
| 3 | Barbados | | Jamaica | Jamaica | Jamaica |
| 4 | Antigua and Barbuda | | Antigua and Barbuda | Antigua and Barbuda | Antigua and Barbuda |
| 5 | Sao Tome and Principe | | Belize | Saint Vincent and the Grenadines | Dominica |
| 6 | Saint Vincent and the Grenadines | | Dominica | Barbados | Saint Vincent and the Grenadines |
| 7 | Dominica | | Barbados | Dominica | Barbados |
| 8 | Belize | | Saint Vincent and the Grenadines | Sao Tome and Principe | Sao Tome and Principe |

­­­

Appendix S9. Jaccard similarity in the top priorities across the scenarios. Debt to GDP 80 scenarios are excluded from this comparative analysis due to not having a large enough pool of qualifying candidates. See Table S5.4 for results. Bold comparisons are results for the sensitivity tests when shipping was treated as an abatable threat (T1) versus as an unabatable threat (T2).

|  | All EEZ T1 | All EEZ T2 | All Reef T1 | All Reef T2 | GDP 40 EEZ T1 | GDP 40 EEZ T2 | GDP 40 Reef T1 | GDP 40 Reef T2 | GDP 60 EEZ T1 | GDP 60 EEZ T2 | GDP 60 Reefs T1 | GDP 60 Reefs T2 |
| --- | --- | --- | --- | --- | --- | --- | --- | --- | --- | --- | --- | --- |
| All EEZ T1 |  | **0.53** | 0.43 | 0.44 | 0.21 | 0.11 | 0.11 | 0.11 | 0.11 | 0.11 | 0.05 | 0.05 |
| All EEZ T2 |  |  | 0.25 | 0.47 | 0.21 | 0.18 | 0.18 | 0.18 | 0.18 | 0.11 | 0.18 | 0.11 |
| All Reef T1 |  |  |  | **0.64** | 0.29 | 0.25 | 0.33 | 0.33 | 0.53 | 0.11 | 0.11 | 0.11 |
| All Reef T2 |  |  |  |  | 0.41 | 0.37 | 0.47 | 0.47 | 0.16 | 0.16 | 0.22 | 0.16 |
| GDP 40 EEZ T1 |  |  |  |  |  | **0.53** | 0.69 | 0.57 | 0.35 | 0.29 | 0.29 | 0.22 |
| GDP 40 EEZ T2 |  |  |  |  |  |  | 0.54 | 0.54 | 0.25 | 0.18 | 0.18 | 0.11 |
| GDP40Reef T1 |  |  |  |  |  |  |  | **0.67** | 0.25 | 0.25 | 0.33 | 0.25 |
| GDP40Reef T2 |  |  |  |  |  |  |  |  | 0.25 | 0.25 | 0.33 | 0.25 |
| GDP 60 EEZ T1 |  |  |  |  |  |  |  |  |  | **0.66** | 0.33 | 0.25 |
| GDP 60 EEZ T2 |  |  |  |  |  |  |  |  |  |  | 0.33 | 0.43 |
| GDP60Reefs T1 |  |  |  |  |  |  |  |  |  |  |  | **0.66** |
| GDP60Reefs T2 |  |  |  |  |  |  |  |  |  |  |  |  |
